# Double-negative feedback between S-phase cyclin-CDK and CKI generates abruptness in the G1/S switch

**DOI:** 10.3389/fphys.2012.00459

**Published:** 2012-12-06

**Authors:** Rainis Venta, Ervin Valk, Mardo Kõivomägi, Mart Loog

**Affiliations:** Institute of Technology, University of TartuTartu, Estonia

**Keywords:** Cdk1, CDK, CKI, cyclin-dependent kinases, Sic1, G1/S, switch, cell cycle

## Abstract

The G1/S transition is a crucial decision point in the cell cycle. At G1/S, there is an abrupt switch from a state of high cyclin-dependent kinases (CDK) inhibitor (CKI) levels and low S-phase CDK activity to a state of high S-phase CDK activity and degraded CKI. In budding yeast, this transition is triggered by phosphorylation of the Cdk1 inhibitor Sic1 at multiple sites by G1-phase CDK (Cln1,2-Cdk1) and S-phase CDK (Clb5,6-Cdk1) complexes. Using mathematical modeling we demonstrate that the mechanistic basis for the abruptness of the G1/S transition is the highly specific phosphorylation of Sic1 by S-phase CDK complex. This switch is generated by a double-negative feedback loop in which S-CDK1 phosphorylates Sic1, thus targeting it for destruction, and thereby liberating further S-CDK1 from the inhibitory Sic1-S-CDK1 complex. Our model predicts that the abruptness of the switch depends upon a strong binding affinity within the Sic1-S-CDK inhibitory complex. *In vitro* phosphorylation analysis using purified yeast proteins revealed that free Clb5-Cdk1 can create positive feedback by phosphorylating Sic1 that is bound in the inhibitory complex, and that Sic1 inhibits Clb5-Cdk1 with a sub-nanomolar inhibition constant. Our model also predicts that if the G1-phase CDK complex is too efficient at targeting Sic1 for destruction, then G1/S becomes a smooth and readily reversible transition. We propose that the optimal role for the G1-phase CDK in the switch would not be to act as a kinase activity directly responsible for abrupt degradation of CKI, but rather to act as a priming signal that initiates a positive feedback loop driven by emerging free S-phase CDK.

## Introduction

The major cell cycle transitions are triggered by phosphorylation or dephosphorylation of the targets of cyclin-dependent kinases (CDKs)(Morgan, 2007). At the transition from G1- to S-phase, the S-phase promoting signal is generated by downregulation of CDK inhibitors (the CKIs) that suppress the activity of the S-phase CDK (Peter, 1997). In budding yeast, the CKI acting at the G1/S transition, the protein Sic1, is simultaneously both an inhibitor and a substrate of Cdk1. At the onset of S-phase Sic1 is phosphorylated by Cdk1, thereby generating two “diphosphodegrons” containing two properly spaced phosphates that are recognized by the Cdc4-SCF ubiquitin ligase (Hao et al., 2007). Ubiquitination by Cdc4-SCF targets Sic1 for proteolysis via the proteasome pathway (Verma et al., 1997b).

Sic1 was first identified as a substrate of Cdk1 by immunoprecipitation (Reed et al., 1985; Wittenberg and Reed, 1988). Later, it was shown to be an inhibitor of Clb-Cdk1 complexes that regulates cell cycle progression at the M/G1 and G1/S transitions (Mendenhall, 1993; Donovan et al., 1994). Transcription of *SIC1* starts in late mitosis and its protein levels increase until the end of G1 phase, followed by a rapid turnover at the G1/S transition (Schwob et al., 1994; Verma et al., 1997b). The signal for the degradation was proposed to be the multisite phosphorylation of Sic1 by G1-specific Cln1,2-Cdk1 complexes. This is possible because, in contrast to S- and M-phase specific Clb-Cdk1s, Cln-Cdk1s are not inhibited by Sic1 (Verma et al., 1997a,b; Nash et al., 2001). The destruction of Sic1 was shown to release the Clb5,6-Cdk1 complexes required to initiate DNA replication (Schwob and Nasmyth, 1993; Schwob et al., 1994; Schneider et al., 1996).

Recently, we showed that the Cdk1-dependent phosphorylation of Sic1 at multiple sites occurs in a specific sequence that forms a semi-processive phosphorylation cascade. This cascade is mediated by Cks1, the phosphate binding adaptor subunit of Cdk1 (Koivomagi et al., 2011a). The S-phase promoting complex Clb5-Cdk1 was shown to be able to efficiently phosphorylate all the crucial sites on Sic1 that were required for generation of phosphodegrons. On the other hand, the G1-specific Cln2-Cdk1 complex, while still capable of phosphorylating Sic1, was much less efficient at transferring phosphates to the critical phosphodegron residues at the end of the cascade. Importantly, Cln-Cdk1 complexes alone were not able to cause the degradation of Sic1 *in vivo*. This led us to propose that positive feedback, arising from the emerging free Clb5,6-Cdk1 complexes, is responsible for the degradation of Sic1, while the Cln1,2-Cdk1 complexes play an auxiliary role as a primer of the phosphorylation cascade.

The relative effectiveness of either Clns or Clbs as the driving force of the G1/S switch depends on several factors, including relative rates of Sic1 phosphorylation and the strength of inhibition of Clb complexes by Sic1. Therefore, to understand the switch, it is crucial to combine quantitative biochemical analysis of these parameters with a modeling approach to evaluate the optimal parameters with respect to the dynamic qualities of the switch. To function as a sharply defined transition point between different cellular states, the CKI- and CDK-controlled G1/S transition should possess both ultrasensitivity and high degree of irreversibility. An ultrasensitive signal-response filters noise by setting a sharp threshold for switch activation and being non-responsive to small signal fluctuations. An abrupt transition is also important for coherence in the activation of S-phase processes and regulatory loops (Skotheim et al., 2008). For example, a gradual transition could create a window in which enough Cdk1 activity exists to fire some origins of replication but not enough activity to fully inactivate all the origins and thereby prevent genotoxic reinitiation. A high degree of irreversibility would also ensure that transient decreases of stimulus would not reverse the switch and interrupt processes that have already initiated.

A sharp transition threshold and irreversibility can be achieved by creating bistability in the switching system (Novak et al., 2007; Domingo-Sananes and Novak, 2010; Zhang et al., 2011). Bistable switches have been described for cell cycle transitions (Ferrell et al., 2009; Trunnell et al., 2011), and the double-negative feedback system of the CKI/CDK module governing the G1/S transition is a potential source of bistability. Alternatively, delayed accumulation of multiply phosphorylated forms of Sic1 has been proposed as a distinct mechanism to create ultrasensitivity at the G1/S transition (Nash et al., 2001), and also for other switches (Kim and Ferrell, 2007; Takahashi and Pryciak, 2008; Harvey et al., 2011; Trunnell et al., 2011; Lu et al., 2012). Again, it is not clear which of the alternative mechanisms provides the optimal solution in the CKI/CDK system involving two different cyclin-CDK complexes and a CKI.

In the present study we analyzed the optimal range and combinations of some of the kinetic parameters governing the G1/S switch. Although a number of parameters may have an impact on the switch, including protein synthesis rates, ubiquitinylation rates, proteolysis rates, and transcription factor (SBF/MBF) activation rates, in this study we focus on the analysis of parameters that control the mutual counteraction of CDK and CKI. We used a minimal model containing both G1- and S-phase CDK complexes together with the CKI, which is an inhibitor of the S-phase-CDK. For the model simulations we used our experimentally validated range of inhibition and phosphorylation parameters and realistic concentrations based on experimental data obtained from the literature. The results provide several important predictions about the parameter ranges that allow for bistability of the system, as well as the realistic dynamic paths of the G1/S switch. The conclusions based on these simulations lay out a general framework for understanding the CDK/CKI switches in eukaryotes.

## Results

### Sic1 inhibits Clb5-Cdk1 stoichiometrically, with subnanomolar K_*i*_

**Figure 1:**
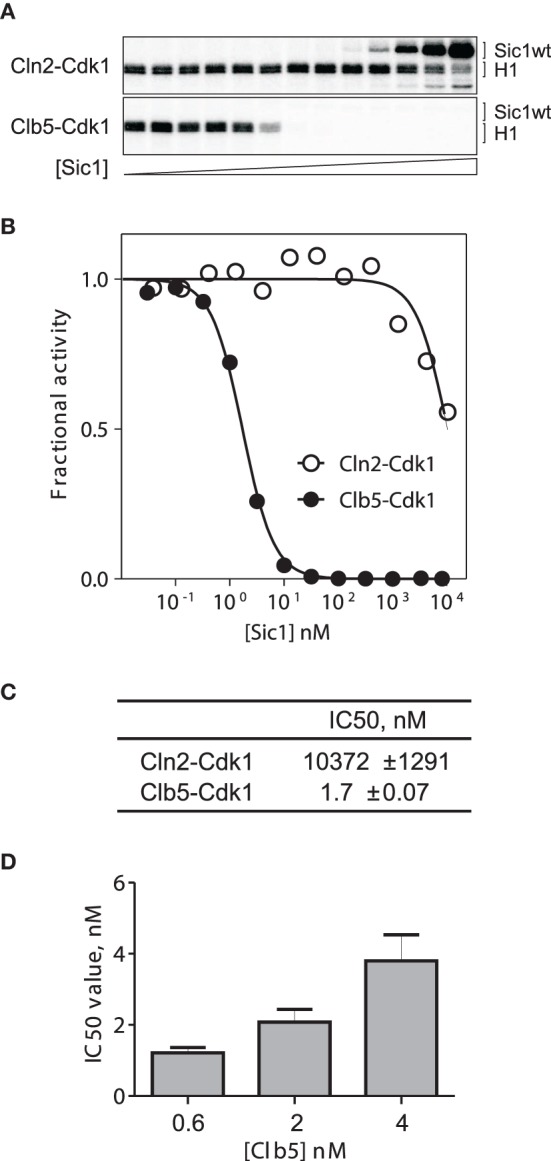
**Inhibition of Clb5-Cdk1 and Cln2-Cdk1 by Sic1. (A)** Autoradiograph of a Clb5-Cdk1 and Cln2-Cdk1 inhibition assay. Sic1 is extensively phosphorylated by Cln2-Cdk1 whereas no detectable phosphorylation is seen with Clb5-Cdk1. **(B)** Inhibition curves for Clb5-Cdk1 and Cln2-Cdk1 are presented as the initial rates of histone H1 phosphorylation in the presence of different concentrations of Sic1. **(C)** Calculated IC50 values from the experiment in panel **(A)**. **(D)** The values of IC50 change in parallel with Clb5-Cdk1concentrations when both Sic1 and the kinase are varied in the picomolar or low nanomolar ranges.

One of the important parameters controlling the G1/S switch is the strength of the inhibition of Clb5/6-Cdk1 complexes by Sic1. To quantitatively measure this inhibition we performed an *in vitro* phosphorylation and inhibition assay with purified Sic1 and cyclin-Cdk1 complexes. Figures 1A–C presents the dose-response curves showing the effect of different concentrations of Sic1 on the initial velocity of histone H1 phosphorylation by Cln2-Cdk1 and Clb5-Cdk1. The IC50 value for Clb5-Cdk1 was 1.7 nM, which places Sic1 into the category of tight stoichiometric inhibitors. As expected, the G1 complex Cln2-Cdk1 was not inhibited by Sic1, and an effect was apparent only in the micromolar range that corresponds to the K_M_ value for the phosphorylation of a non-inhibitory version of Sic1 (data not shown). This suggests that Sic1 only inhibits Cln2-Cdk1 as a competitive substrate.

The experiment presented in Figures 1A–C contained 500 μM ATP and 2.5 μM Histone H1. The K_M,ATP_ = 70 μM, and the value for K_M,H1_ is above 10 μM (our unpublished data). Therefore, the obtained value of IC50 is a valid estimate of the apparent inhibition constant for substrate phosphorylation at saturating and nearly physiological ATP concentration. However, the experiment presented in Figures 1A–C was performed at low Clb5-Cdk1 levels (1.5–2 nM) that are used in our standard assay conditions. Since the estimated IC50 value was in the same concentration range as the enzyme, the inhibition constant may prove to be even lower. When the enzyme concentration was lowered or raised, the IC50 value changed as well (Figure 1D). This suggests that we can give an estimate for the apparent K_*i*_ value to be in the range of 100 pM to 1 nM. The exact value of such a constant is difficult to determine using available methods. This extremely tight inhibition complex should exhibit a very low off-rate, which is an important factor in modeling the Sic1 phosphorylation and degradation switch, as discussed below.

### Sic1 is efficiently phosphorylated within the Sic1/Clb5-Cdk1 complex

The dynamics of the switch are also shaped by the rates of Sic1 phosphorylation by Cln1,2-Cdk1 and Clb5,6-Cdk1. We have previously shown that both Clb5-Cdk1 and Cln2-Cdk1 efficiently phosphorylate the N-terminal part of the truncated non-inhibitory version of Sic1 (Sic1ΔC) (Koivomagi et al., 2011a). Substrate specificity was shown to be gained by an interaction between a hydrophobic docking site in Clb5 and RXL motifs in Sic1, and in the case of Cln2-Cdk1 via a novel hydrophobic LLPP-docking motif in Sic1 (Koivomagi et al., 2011b; Bhaduri and Pryciak, 2011; Koivomagi and Loog, 2011). Furthermore, by detailed mapping of the paths of the multisite phosphorylation cascades we found that the Clb5 specificity toward different sites, including the pair S76/S80, which form the critical diphosphodegrons at the end of the cascade, was much more pronounced compared to Cln2. These observations led to the conclusion that although Cln2-Cdk1 can phosphorylate Sic1, it cannot trigger Sic1 degradation without the help of Clb5-Cdk1. This conclusion was supported by *in vivo* experiments showing that, in the absence of B-type cyclin (Clb) activity, the Clns alone were not able to cause the degradation of Sic1. Thus, Clb-Cdk1 activity is the driving force of Sic1 degradation at the G1/S transition. This underscores the likely importance of a double-negative feedback mechanism whereby Clb5-Cdk1 phosphorylates Sic1, leading to its destruction and thereby releasing further active Clb5-Cdk1 from the inhibitory Sic1-Clb5-Cdk1 complex.

**Figure 2:**
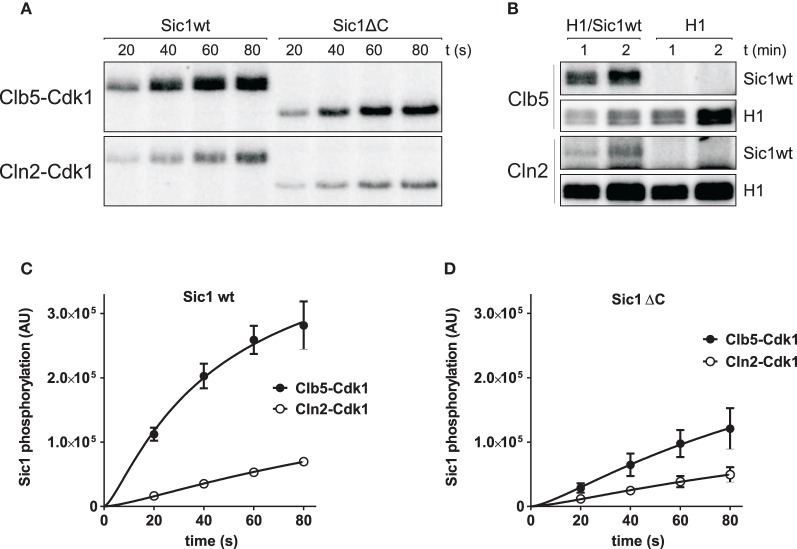
**Sic1 is efficiently phosphorylated within the Sic1/Clb5-Cdk1 complex, when an excess of Clb5-Cdk1 is added.** Assays with Cln2-Cdk1 complexes are included for comparison. **(A)** Autoradiography showing the phosphorylation of purified Sic1wt and Sic1ΔC by Clb5-Cdk1 and Cln2-Cdk1 complexes using 15 nM Sic1 and 30 nM cyclin-Cdk1 complexes. **(B)** The activity of Clb5-Cdk1 was determined using 2.5 μM histone H1, with and without 15 nM Sic1wt in the assay mixture. **(C,D)** Quantification of the experiment presented in panel **(A)**. For comparison, phosphorylation rates were normalized using cyclin-Cdk1 activity units obtained in panel **(B)**.

This model requires that Clb5-Cdk1 can efficiently phosphorylate Sic1 bound to the inhibited Clb5-Cdk1 complex. To test this, we performed a Sic1 phosphorylation assay using partly inhibited Clb5-Cdk1—that is, we tested the ability of Clb5 to phosphorylate a Sic1-Clb5-Cdk1 complex (Figures 2A–D). Additionally, the phosphorylation of non-inhibitory Sic1ΔC was assayed in the same conditions. In these assays, 30 nM Clb5-Cdk1 or Cln2-Cdk1 was mixed with 15 nM Sic1. To quantify the exact fraction of uninhibited Clb5-Cdk1 activity, a control substrate histone H1 was included in a separate assay (Figure 2B). Clb5-Cdk1 phosphorylated both forms of Sic1 efficiently and at a considerably higher rate than Cln2-Cdk1. The rate of phosphorylation of the full length Sic1 was similar to that of Sic1ΔC for both Cln2 and Clb5. These data indicate that the N-terminus of Sic1 is freely accessible for phosphorylation when Sic1 is bound in the inhibitory complex, and that emerging free Clb5-Cdk1, released from the inhibitory complex as Sic1 is degraded, could accelerate Sic1 phosphorylation and degradation, thus creating a double-negative feedback.

We have also observed that a low level of intramolecular Sic1 phosphorylation takes place within the inhibitory Sic1-Clb5-Cdk1 complex during its formation (data not shown). It is partially also the likely reason why the full length Sic1 showed slightly faster phosphorylation rate compared to Sic1ΔC (Figures 2C,D). Indeed, structural modeling studies do not exclude the possibility that the active site of Cdk1 may be partly accessible during the inhibitor binding (Barberis et al., 2005a,b; Barberis, 2012a,b). The possible role of these phosphorylation events in the switch is currently under investigation.

### The model of the CDK/CKI-controlled G1/S switch

**Figure 3:**
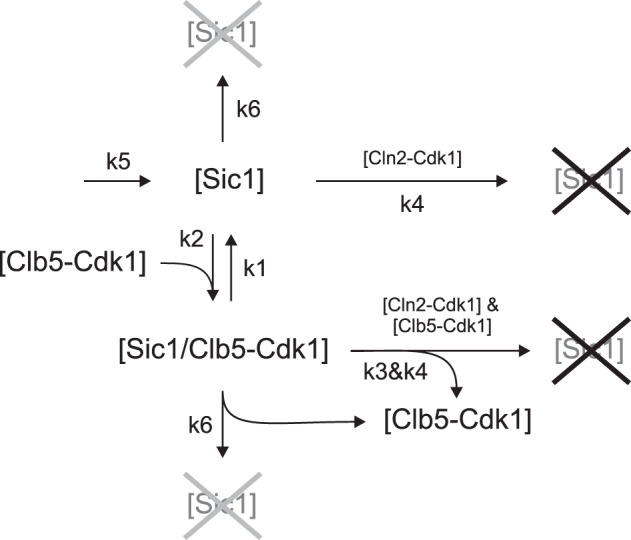
**Minimal model of the G1/S switch.** Parameters used for diagrams and simulations presented in the figures below are listed in Tables 1 and 2. Black crosses designate the phospho-dependent degradation of Sic1 via the SCF-proteasome pathway. Gray crosses designate basal degradation of Sic1.

To quantitatively analyze the impact of the inhibition strength and the phosphorylation rates on the abruptness and the degree of irreversibility of the G1/S switch, we constructed a minimal model of Sic1 phosphorylation and degradation (Figure 3, Table 1). As a first step, we analyzed the steady state output of the model. This is important to understand the system in general and to analyze the transition thresholds for the conditions (e.g., slow growth, the arrest, or stress) when the degree of irreversibility of the switch may become especially important. As a second step we analyze the behavior of the switch in the normal dynamic circumstances of the cell cycle.

**Table 1:** **Equations describing the minimal model of the G1/S switch**.

**Figure 4:**
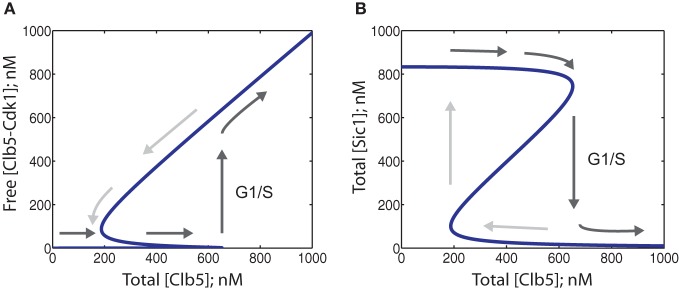
**Phase diagrams showing the bistability of the system at steady state.** Parameter values are provided in Table 1. The dark gray arrows show possible paths for a gradual increase of free Clb5-Cdk1 while entering the S-phase, and possible reversible paths (light gray arrows) in the event of stochastic decreases in Clb5 levels. **(A)** Phase diagram showing the dependence of free Clb5-Cdk1 activity on total Clb5-Cdk1 levels. When a threshold is reached, the system switches itself into the state corresponding to S-phase, as indicated by the upward pointing arrow. **(B)** Phase diagram for the same system with the same parameter values as in panel **(A)**, showing the dependence of total Sic1 concentration on total Clb5-Cdk1. The threshold level for a drop of Sic1 corresponds to the same threshold level of total Clb5-Cdk1 that triggers the jump in steady state levels of free Clb5-Cdk1 in panel **(A)**.

$\frac{dy_{1}}{dt}=k_{5}-k_{2}y_{1}y_{2}+k_{1}y_{3}-k_{6}y_{1}-k_{4}y_{1}y_{4}$
$\frac{dy_{2}}{dt}=k_{3}y_{2}y_{3}-k_{2}y_{1}y_{2}+k_{1}y_{3}+k_{6}y_{3}+k_{4}y_{3}y_{4}$
$\frac{dy_{3}}{dt}=k_{2}y_{1}y_{2}-k_{3}y_{2}y_{3}-k_{1}y_{3}-k_{6}y_{3}-k_{4}y_{3}y_{4}$
*y*_1_ = [Sic1_FREE_]
*y*_2_ = [Clb5 − Cdk1_FREE_]
*y*_3_ = [Sic1/Clb5 − Cdk1]
*y*_4_ = [Cln2 − Cdk1] =constant at 200 nM
Conservation equations:
*y*_2_ = [Clb5 − Cdk1_TOTAL_] − *y*_3_
*y*_1_ = [Sic1_TOTAL_] − *y*_3_
Kinetic constants (the values presented below are those used in Figure 4. Parameter values that are varied in other figures are given in the legends or on the figure panels):
*k*_1_ = 0.01 min^−1^; dissociation rate of the inhibitory complex
*k*_2_ = 0.1 nM^−1^ × min^−1^; association rate of the inhibitory complex
*k*_3_ = 0.001 nM^−1^ × min^−1^; the net rate constant for Clb5-dependent phosphorylation and degradation of Sic1
*k*_4_ = 0.00001 nM^−1^ × min^−1^; the net rate constant for Cln2-dependent phosphorylation and degradation of Sic1
*k*_5_ = 10 nM × min^−1^; the basal synthesis rate of Sic1
*k*_6_ = 0.01 min^−1^; the basal degradation rate of Sic1

**Figure 5:**
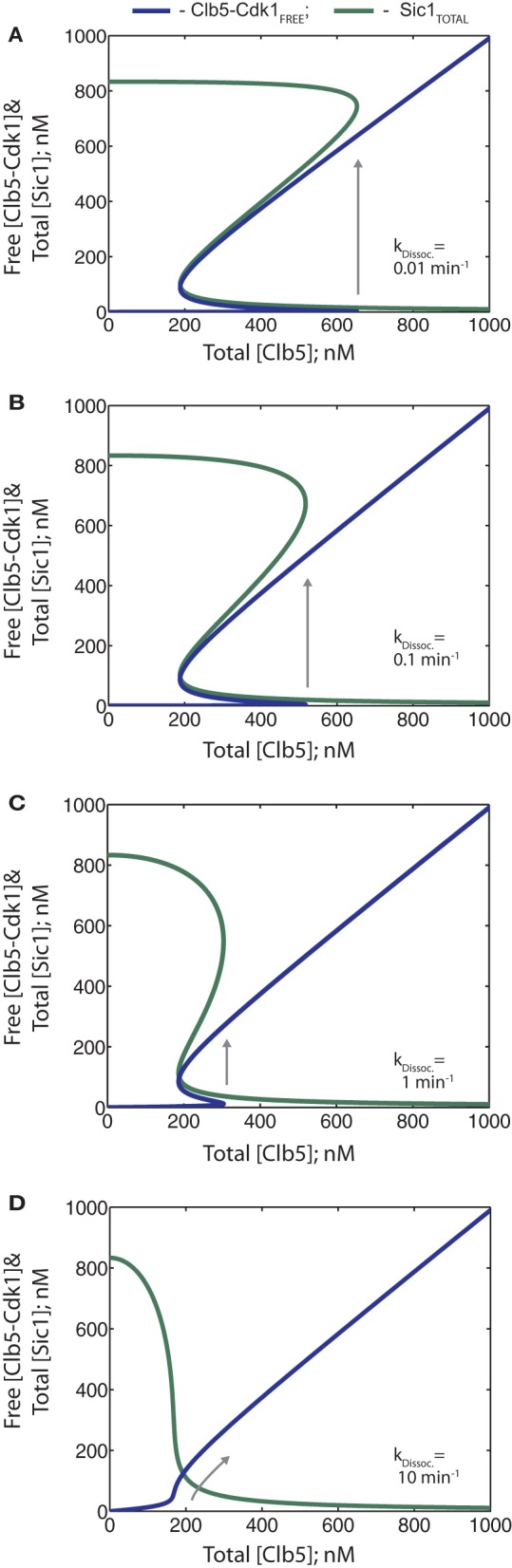
**(A–D)** The steady state phase diagrams showing the effect of the inhibition strength on the bistability of the G1/S switch. The dissociation rates of the inhibitory complex were increased in 10-fold increments from 0.01 to 10 min^−1^ as indicated. In the model, these values correspond to K_*i*_ values of 0.1–100 nM. Arrows indicate the paths of the system at the G1/S transition.

**Figure 6:**
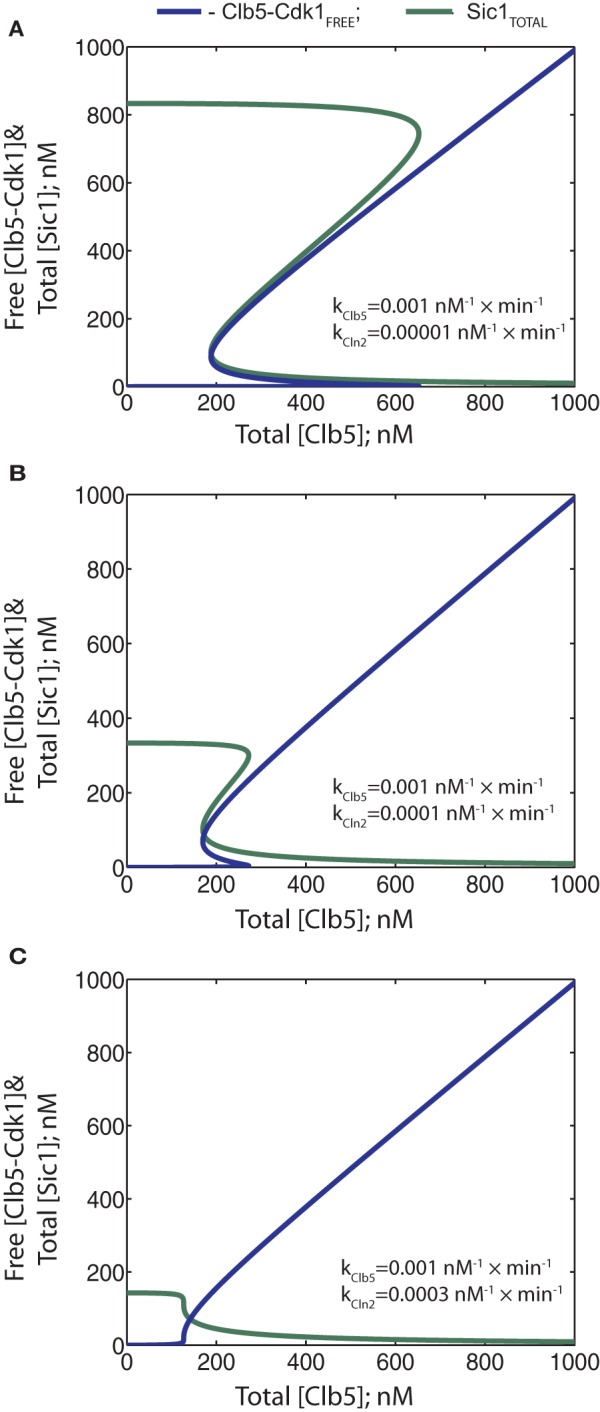
**(A–C)** Phase diagrams showing the effect of changes in the relative ability of Cln2-Cdk1 to directly cause the degradation of Sic1. The rate constant values for Cln2-Cdk1 were gradually increased as indicated.

**Figure 7:**
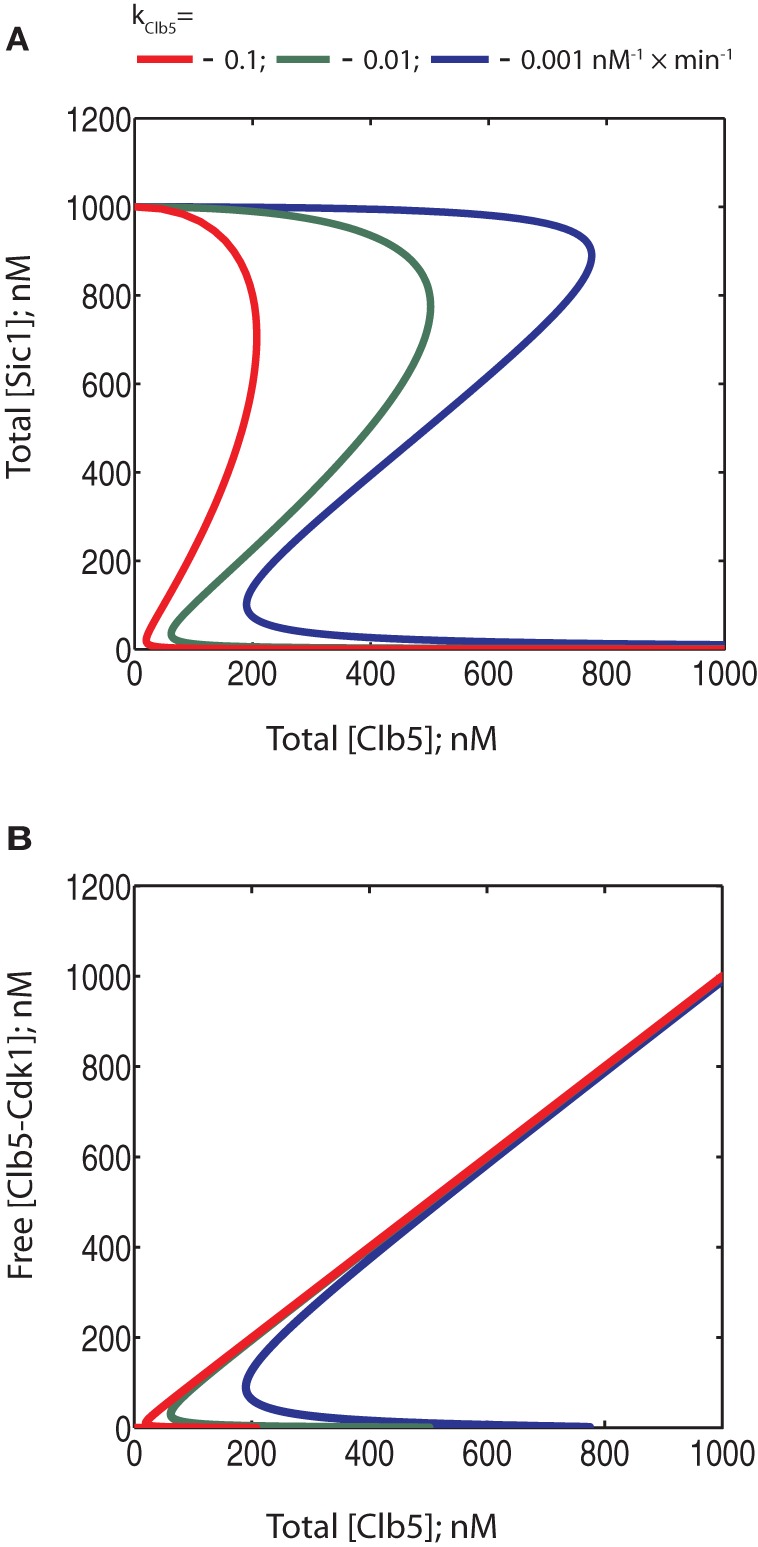
**Phase diagrams showing the potential effect of Cln2-dependent priming phosphorylation on the bistability of the system.** The rate constant value of 0.001 nM×min^−1^ for Clb5-Cdk1 exemplifies a system with no Cln2-dependent priming while increasing values of this constant mimic increased levels of Cln2-dependent priming effect. **(A)** Phase diagrams showing the dependence of free Clb5-Cdk1 activity on total Clb5-Cdk1 levels. **(B)** Phase diagrams showing the dependence of total Sic1 concentration on total Clb5-Cdk1. The color coding is the same for both panels.

The description of the system is presented in Figure 3. For the phase diagrams presented in Figures 5–7, the steady-state solutions for the dependencies between Sic1_TOTAL_ and Clb5-Cdk1_TOTAL_ and Clb5-Clb5-Cdk1_FREE_ and Clb5-Cdk1_TOTAL_ were derived.

To simplify our model and avoid equation systems that lack steady state solutions, we did not include the multisite phosphorylation steps, but considered the net phosphorylation and degradation process as a single step. This was based on our experimental data showing that accumulation of multiply phosphorylated species is a rapid, semi-processive process *in vitro*, and that multiply phosphorylated forms of Sic1 do not accumulate *in vivo* after cells are released from an α-factor arrest (Koivomagi et al., 2011a). The latter observation suggests that Sic1 degradation is rapid compared to phosphorylation. Therefore, we can consider the rate of phosphorylation and degradation to be a single value that is mostly determined by phosphorylation rates. Also, due to the negligible accumulation of multi-phosphorylated forms of Sic1 and prompt degradation in response to increasing Cdk1 activity *in vivo*, we omitted the counteracting phosphatase from the model. This does not mean that the phosphatase does not take part in the process but that, for simplicity, its effect was included in the net rate constant of the whole process. The first of the three ordinary differential equations of the model describe free Sic1 levels, including the association and dissociation of the inhibitory complex and Cln2-dependent phosphorylation/degradation (Table 1). The second equation describes the dynamics of free Clb5 concentration, which is controlled by the association-dissociation rates of the inhibitory complex and also by the fast release of Clb5-Cdk1 from the inhibitory complex upon Sic1 destruction (Verma et al., 2001). The third equation describes the concentration changes of the inhibitory complex. The conservation equations define the total levels of Clb5 and Sic1. Finally, the model includes slow basal synthesis and degradation of Sic1. This was based on the finding that although the major Swi5-induced transcription of *SIC1* peaks at the exit from mitosis (Schwob et al., 1994), *SIC1* transcription is still observed throughout the entire cell cycle (Knapp et al., 1996; Aerne et al., 1998). Taken together, although considerably simplified, this minimal model contains the basic and universal core elements of a switch containing a double-negative feedback between a CKI and a CDK. Additionally, the system contains a second CDK complex (the equivalent of Cln2-Cdk1) that is not inhibited by CKI but acts as an initiator of the switch.

The values for the phosphorylation rate constants were derived from our experimentally determined rates of the phosphorylation of Sic1 and other specific model substrates by cyclin-Cdk1 complexes (Loog and Morgan, 2005; Koivomagi et al., 2011a,b). A K_M_ value of 3.3 μM and a k_cat_ value of 73 min^−1^ were determined for Clb5-Cdk1-dependent phosphorylation for Sic1 (data not shown). Considering that there are about six to seven phosphorylation steps, and only a slight phosphatase counteraction, the net rate constant for the process of Clb5-dependent phosphorylation (k_Clb5_) and degradation of Sic1 was taken to be 0.001 nM^−1^ × min^−1^. Although Cln2-Cdk1 showed only a 3–4-fold lower rate for net phosphorylation of Sic1 in the Sic1-Clb5-Cdk1 complex (Figure 2), it was shown to have a considerably weaker ability to phosphorylate the priming site T33, and also, to finalize the phosphorylation cascade, thus creating a di-phosphodegron pS76/pS80 (Koivomagi et al., 2011a). Therefore, the net rate constant for Cln2-dependent phosphorylation (k_Cln2_) was initially taken to be 0.00001 nM^−1^ × min^−1^, two orders of magnitude lower than the corresponding value for Clb5-Cdk1. The importance of this difference on the properties of the switch will be further analyzed below. Note, as explained above, that these rate constant are the net constants for both phosphorylation and degradation.

The parameters defining the inhibitory interaction between Sic1 and Clb5-Cdk1 were derived from the inhibition experiment presented in Figure 1. Assuming that the diffusion-limited association rate of an average protein–protein interaction that involves an intrinsically disordered binding partner is in the range of 10^7^ to 10^8^ M^−1^ × min^−1^, then a K_D_ of a few hundred pM would yield a dissociation rate for the Sic1-Clb5-Cdk1 inhibitory complex in the range of 10^−3^ to 10^−2^ min^−1^. The effect of varying this parameter will be studied in the context of the switch. The basal synthesis and degradation rates were taken to be relatively slow, and for the phase diagrams below these values were chosen to give a steady-state concentration of Sic1 of 1 μM in the absence of cyclin-Cdk1 complexes.

When the steady-state solution of this ODE system is presented as a plot of total Clb5 levels vs. the fraction of free Clb5 at a fixed Cln2 concentration, a steep hysteresis loop can be observed, indicating that the system is a bistable switch (Figure 4A). The levels of total Sic1 vs. total Clb5 also exhibit hysteresis (Figure 4B). These diagrams show the two states of the switch separated by two saddle node bifurcations. The state with high total Sic1 and low free Clb5-Cdk1 corresponds to G1, and the state with low total Sic1 and high levels of free Clb5-Cdk1 corresponds to S-phase. If the cell is in G1 and the total Clb5 concentration is gradually rising, the phase paths, as shown by the arrows, reach a threshold upon which there is a jump in free Clb5-Cdk1 steady-state value and a drop in Sic1 levels. This is the on-switch for S-phase. This bistable system sets a high threshold for Clb5, thereby filtering noise, before a sharp transition that ensures an abrupt and coherent start of S-phase processes. Also, the switch between the two states is highly irreversible and resistant to transient fluctuations in Clb5 levels. Once activated, it is hard to reverse the switch, as it would take a several-fold drop in Clb5 concentration before Sic1 can re-accumulate to switch the system back to the G1 state.

### Tight inhibition of Clb5-Cdk1 by Sic1 and efficient phosphorylation of Sic1-Clb5-Cdk1 by free Clb5-Cdk1 are crucial for setting a discreet threshold for the G1/S transition

To understand the basic features of the switch and to determine the optimal balance of crucial parameters that are required to maintain the bistable nature of the system, we studied the effect of inhibition strength and phosphorylation rates on the behavior of the phase diagrams. In the diagrams presented in Figures 5A–D, inhibition strength was varied by introducing different values for the dissociation rate of the inhibitory complex. As the dissociation rate increased, the bistable behavior of the system gradually weakened and the switch evolved to a smooth transition that can easily slide back and forth (Figure 5D). This kind of a behavior would potentially be very harmful for a transition like G1/S: only a slight backward deviation of the total Clb5 levels would reverse the cell to G1, interrupting processes like the initiation of replication, and removing the re-replication block. Thus, our model predicts that between a K_*i*_ of 10–100 nM the on-threshold continues to decrease, and eventually the system loses its hysteresis, as well as its switch-like response (Figures 5C,D). In this range, Sic1 becomes a readily overcome barrier for the accumulating Clb5.

Next, in the diagrams presented in Figure 6, we explored the effect of relative phosphorylation rates. To understand the impact of the experimentally determined strikingly weaker ability of Cln2-Cdk1 to phosphorylate the critical output degron sites in Sic1, compared to Clb5-Cdk1 (Koivomagi et al., 2011a), we first varied the Cln2-dependent phosphorylation rate relative to a fixed Clb5-dependent rate (Figures 6A–C). Importantly, steeper hysteresis was observed when the Cln2-dependent phosphorylation rate was considerably lower compared to the rate of Clb5. Increasing the relative Cln2-dependent rate resulted in less hysteresis, lowered initial Sic1 levels, and lowered the Clb5 threshold for the transition (Figures 6A,B). Thus, the timing of the transition could potentially be tuned by Cln2 activity. However, when Cln2 specificity toward Sic1 approaches the specificity of Clb5, the requirement for Clb5 signal almost ceases to exist (Figure 6C). Similarly, as in the case of a weak K_*i*_ (above), the threshold of Total (Clb5) decreased and the discontinuity of the Clb5-Cdk1 switch was lost. Thus, both tight inhibition and cyclin specificity are important factors for an abrupt transition and a high degree of irreversibility. Interestingly, the effect of Cln2 specificity on bistability was more pronounced compared to the effect of an increase in K_*i*_ of the same order.

Thus, in the system described in Figure 3, where both Cln2 and Clb5 are able to send Sic1 for degradation, the signal strength of Cln2 can potentially modulate timing by lowering the threshold of Clb5 required to trigger the switch. However, our recent results indicate that Cln2-Cdk1 cannot phosphorylate the last phospho-degron sites in Sic1 at a sufficient rate to cause degradation *in vivo*. On the other hand, Cln2-Cdk1 can efficiently phosphorylate the initial residues in the Sic1 cascade, and this primes Sic1 for efficient subsequent phosphorylation by Clb5-Cdk1, which will finalize the cascade to send Sic1 to degradation (Koivomagi et al., 2011a). To study how such a priming effect could influence the switch, we gradually raised the phosphorylation rate constant for Clb5 in the absence of Cln2 activity. In this simplified way, the different constants would roughly mimic different levels of Cln2 as a priming kinase (higher rate constants being equivalent to primed Sic1). The diagrams in Figure 7 reveal that, similarly to the scenario in Figure 6, priming by Cln2 would influence the threshold of the switch. However, the abruptness of the transition is not lost within a realistic range of rate constant values for Clb5-Cdk1. Such tuning of the threshold of the switch by priming can act as a mutual control system between the G1-CDK and S-CDK activities. If there is not enough S-phase CDK activity, the Sic1 degradation switch is not triggered. If there are sufficient levels of the S-phase CDK activity, but for some reason the levels of G1-CDK drop, the transition would be delayed. Thus, a priming mechanism has the advantage of connecting the Cln and Clb signals, allowing tuning of decision-making by both pathways. When Cln and Clb signals are independent, there is a possibility that the switch will be triggered before sufficient amounts of Clb5 are accumulated. In the priming model, Sic1 degradation and lifting the barrier from S-phase always requires sufficient levels of Clb5 or other early expressed B-type cyclins.

### Cyclin concentrations and synthesis rates

Although the phase diagrams provide a good general characterization of an ODE model describing a bistable system, they don't necessarily fully reflect the way the system behaves in reality. Some rates could be too slow relative to the others, in which case steady states would not be established. In the present case, the synthesis of Clb5 is fast, with a peak accumulation time of only around 10–20 min (Koivomagi et al., 2011b). Therefore, there is a possibility that relatively slow phosphorylation rates or dissociation rates of the inhibitory complex would not allow the formation of the steady states described by the phase diagrams. To address this concern, we investigated the dynamics of our model. We numerically simulated a modified form of the ODE system (Table 2). We searched the literature to supply realistic synthesis rates and peak concentrations of the cyclins and Sic1. We obtained experimentally estimated endogenous concentrations of Cln1, Cln2, Cln3, Clb5, Clb6, and Sic1. The most detailed studies have been performed by western blotting of ProtA-tagged proteins and by using quantitative calibration of the blotting signals with ProtA standards (Cross et al., 2002). Second, a study presenting a global analysis of protein abundances in yeast provides quantifications based on the western blotting of TAP-tagged proteins (Ghaemmaghami et al., 2003). Using the ProtA-tag method, the estimated average number of Sic1 molecules per diploid cell in asynchronous cultures was reported to be 214. The study using the TAP-tag in haploid cells provided an estimate of 768 copies of Sic1 per cell. To calculate the realistic initial concentration value for Sic1 for the ODE simulations, one needs to take into account the predominantly nuclear localization of Sic1 (Huh et al., 2003). The nuclear volume of haploid and diploid cells is thought to be 2 and 4 fl, respectively (Cross et al., 2002). Thus, the average estimated nuclear concentration of Sic1 in asynchronous cultures would be 363 nM. Since Sic1 is present during only a fraction of the cell cycle, we considered that a reasonable peak concentration would be three times that of the average abundance in asynchronous cells. Therefore, a realistic concentration of Sic1 at its peak in G1 would be around 1 μM. In fact, this value falls into the same range as the steady state value of Sic1 in the absence of Clb5 and Cln2 in the phase diagrams presented in Figure 7.

**Table 2:** **Equation system used for numerical simulation of the time courses at G1/S in Figures 8A–C, 9A–D, and 10A**.

**Figure 8:**
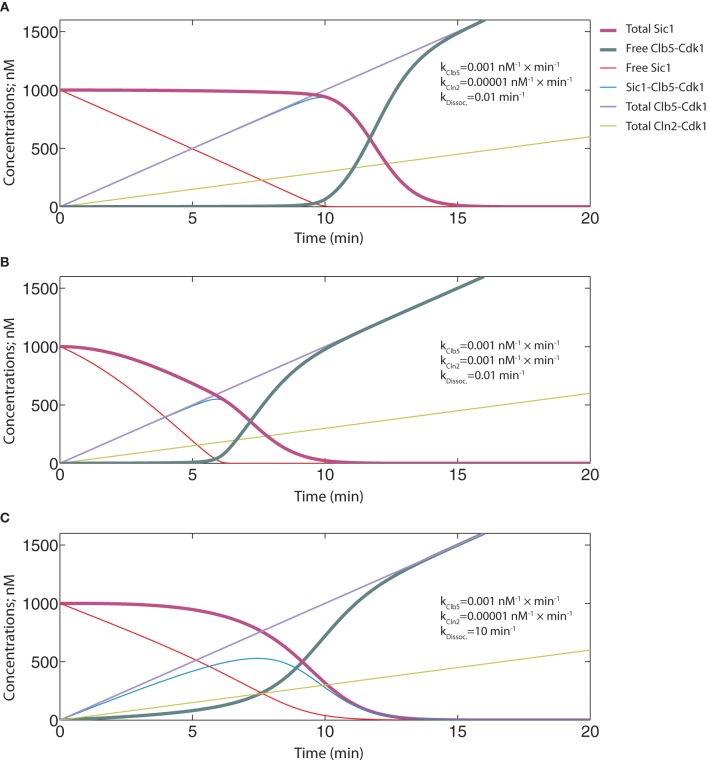
**Time course simulations to analyze the abruptness of the transition.** The equation system and parameter values are provided in Table 2. **(A)** The simulation with the basic set of key parameters for inhibition and phosphorylation, similar to those used in the phase diagrams in Figure 4. **(B)** The effect of faster Cln2-dependent phosphorylation rate on the switch. The rate constant value for Cln2-Cdk1 was taken to be equal to the “k” value of Clb5-dependent phosphorylation. **(C)** The effect of weaker inhibition strength on the switch. The dissociation rate of the inhibitory complex was raised to 10 min^−1^, which in the model corresponds to a K_*i*_ value of 100 nM.

$\frac{dy_{1}}{dt}=-k_{2}y_{1}y_{2}-k_{4}y_{1}y_{4}+k_{1}y_{3}$
$\frac{dy_{2}}{dt}=k_{8}-k_{2}y_{1}y_{2}+k_{3}y_{2}y_{3}+k_{4}y_{3}y_{4}+k_{1}y_{3}$
$\frac{dy_{3}}{dt}=k_{2}y_{1}(y_{6}-y_{3})-k_{3}y_{2}y_{3}-k_{4}y_{3}y_{4}-k_{1}y_{3}$
$\frac{dy_{4}}{dt}=k_{7}$
$\frac{dy_{5}}{dt}=-k_{4}y_{1}y_{4}-k_{3}y_{2}y_{3}-k_{4}y_{3}y_{4}$
$\frac{dy_{6}}{dt}=k_{8}$
*y*_1_ = [Sic1_FREE_]
*y*_2_ = [Clb5 − Cdk1_FREE_]
*y*_3_ = [Sic1/Clb5 − Cdk1]
*y*_4_ = [Cln2 − Cdk1]
*y*_5_ = [Sic1_TOTAL_]
*y*_6_ = [Clb5 − Cdk1_TOTAL_]
The kinetic constants (values presented below are those used in Figure 8A. Parameter values for other figures are given in the legends or on the figure panels):
*k*_1_ = 0.01 min^−1^; dissociation rate of the inhibitory complex
*k*_2_ = 0.1 nM^−1^ × min^−1^; association rate of the inhibitory complex
*k*_3_ = 0.001 nM^−1^ × min^−1^; the net rate constant for Clb5-dependent phosphorylation and degradation of Sic1
*k*_4_ = 0.00001 nM^−1^ × min^−1^; the net rate constant for Cln2-dependent phosphorylation and degradation of Sic1
*k*_7_ = 100 nM × min^−1^; Cln2 synthesis rate
*k*_8_ = 30 nM × min^−1^; Clb5 synthesis rate

The initial values for y1 (Sic1_FREE_) and y5 (Sic1_TOTAL_) in two simulations were 1 μM.

Analogous calculations for Clb5, whose localization is also predominantly nuclear (Shirayama et al., 1999), give peak concentration of 1138 nM (115 nM for Clb6). The cyclins Cln1 and Cln2 are localized both in cytoplasm and in nucleus (Miller and Cross, 2000; Edgington and Futcher, 2001; Landry et al., 2012), and therefore the cell volume for calculating their concentration was taken to be 100 fl for the ProtA study (diploid cells) and 50 fl for the TAP study (haploid cells). Interestingly, the obtained peak values for Cln1 and Cln2 were about 164 and 33 nM, respectively, which is much lower than the peak values for Clb5 and Clb6. This fact would additionally support the model that Cln1,2 may have a less critical role in catalyzing Sic1 degradation compared to relatively abundant Clb5. Cln3 levels were recorded only by the study using ProtA, and their concentration in G1, taking into account the nuclear localization, was in the range of 269 nM.

Based on these calculations and the half-times of accumulation of Cln2 and Clb5, the concentrations of Cln2 and Clb5 in this model were set to rise at 30 and 100 nM per minute, respectively. To obtain a clear and formal picture of the effect of accumulation rates on the dynamics of the switch, a constant linear accumulation was introduced (Table 2). During the short time window of cyclin accumulation, the basal synthesis and degradation rates of Sic1 used in the ODE system for the phase diagrams would be too slow to significantly affect the dynamics of Sic1 degradation. Therefore, these rates were excluded to lower the complexity of the model.

### Tight inhibition and efficient Clb5-dependent Sic1 phosphorylation is important for the abruptness of the switch under realistic dynamic circumstances

The simulation of the ODE system presented in Figure 8A represents a situation with tight inhibition and high k_Clb5_/k_Cln2_ ratio, analogous to the one presented in Figure 4. Although exhibiting a time course with a sigmoidal shape, a fairly slow transition from low to high Clb5-Cdk1 activity level is observed. Thus, although the phase diagrams can provide a general description of the system, the transitions between the alternative states may happen less abruptly. We also investigated the effect of lowering the k_Clb5_/k_Cln2_ ratio. Similarly to the conclusion made based on the phase diagrams above, a relatively faster Cln2-dependent rate decreased the ultrasensitivity of the signal response and slowed the transition (Figure 8B). Also, analogously to the tendency observed in Figure 5, increasing the K_*i*_ value in these dynamic circumstances reduced the abruptness of the switch (Figure 8C).

**Figure 9:**
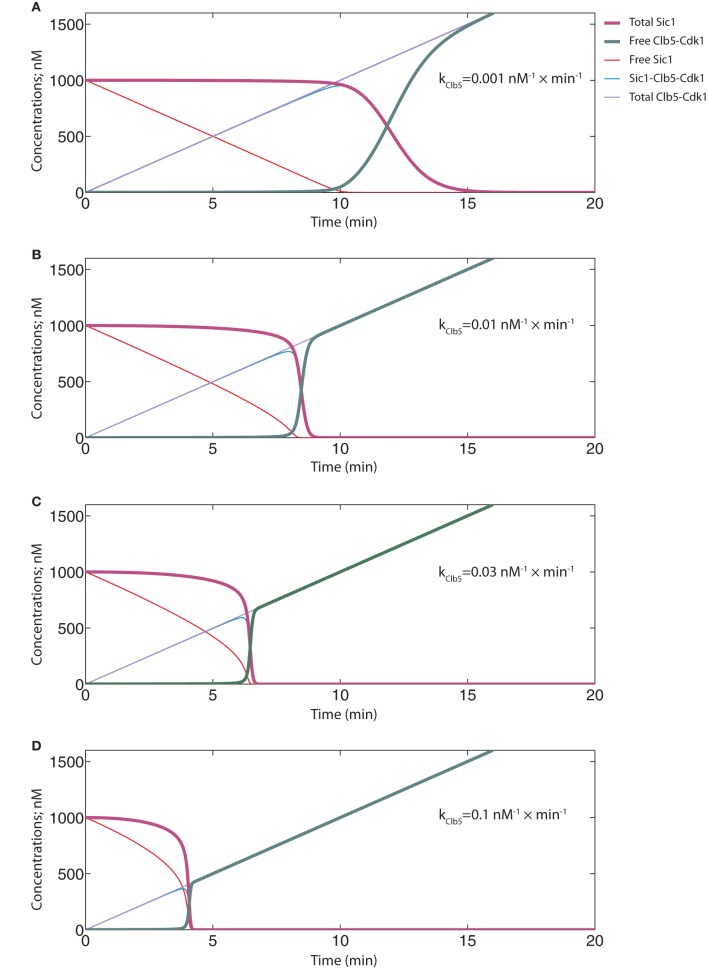
**Time course simulations showing the potential effect of Cln2-dependent priming phosphorylation on the abruptness of the switch.** The equation system and parameter values are provided in Table 2. **(A)** A system with no priming effect in the absence of Cln2. The basal rate constant value of 0.001 nM^−1^ × min^−1^ for Clb5-Cdk1 was used. **(B–D)** Different priming effects were mimicked by using the following “k” values for Clb5-Cdk1: 0.01 nM^−1^ × min^−1^ **(B)**, 0.03 nM^−1^ × min^−1^ **(C)**, and 0.1 nM^−1^ × min^−1^ **(D)**.

If Cln2-Cdk1 was left out of the model and Clb5-Cdk1 had its usual rate constant value of 0.001 nM^−1^ × min^−1^, the system behaved like the one in Figure 8A (k_Cln2_ = 0.0001 nM^−1^ × min^−1^), with free Clb5-Cdk1 only starting to appear when the concentration of total Clb5 surpassed the initial concentration of Sic1 at 1000 nM (Figure 9A). In both of these cases the start of Sic1 degradation corresponds to the point at which Clb5 surpasses stoichiometry with Sic1. The slow Sic1 degradation profile, in this case, shows that the k_Clb5_ value of 0.001 nM^−1^ × min^−1^ alone would not be sufficient to cause the transition to be abrupt. However, in such a situation S-phase would still be initiated with only a few minutes delay. This is in agreement with our experimental observations that the mutation of the Cln1,2-specific docking motif in Sic1 (the LLPP motif) introduced about a 5–10 min delay in Sic1 degradation after the release of the cells from the G1 arrest (Koivomagi et al., 2011a).

When the effect of Cln2 was introduced in the capacity of a primer for Clb5, it was possible to tune the threshold of the transition (Figures 9A–C). In these simulations, different phosphorylation constant values for Clb5 present examples of different priming effects created by Cln2. Importantly, Cln2-dependent priming introduced abruptness to the switch. It is plausible that priming (phosphorylation of sites by Cln2-Cdk1 that Clb5-Cdk1 can subsequently utilize as docking sites for Cks1-dependent finalization of the cascade) creates a mixture of Sic1 forms whose net phosphorylation rate by Clb5 is optimal to cause a rapid decline of Sic1 levels. Indeed, lowering the number of steps in the cascade that Clb5 must pass through would effectively raise the net rate of the Clb5-dependent process. Also, priming of the non-Cks1-dependent slow initial step by another kinase would lessen the burden on Clb5. Interestingly, when the k_Clb5_ value was raised further to 0.1 nM^−1^ × min^−1^, abruptness was retained, but the Clb5 threshold for the G1/S transition would be about three times lower than the Clb5 peak value (Figure 9D). This scenario would create a weak noise barrier and would also lack the potential for a burst activation of S-phase with the full potential of accumulating Clb5. This situation would potentially cause genomic instability, a phenotype characteristic of the *SIC1*Δ strain (Lengronne and Schwob, 2002). Thus, the estimated range of net Clb5-dependent phosphorylation rates of 0.01 nM min^−1^ for the mixture of primed forms of Sic1 is in an ideal window to achieve abruptness while maintaining a noise-filtering threshold. It is conceivable that multisite phosphorylation systems provide wide combinatorial flexibility for tuning and optimization of these kinetic properties during evolution.

**Figure 10:**
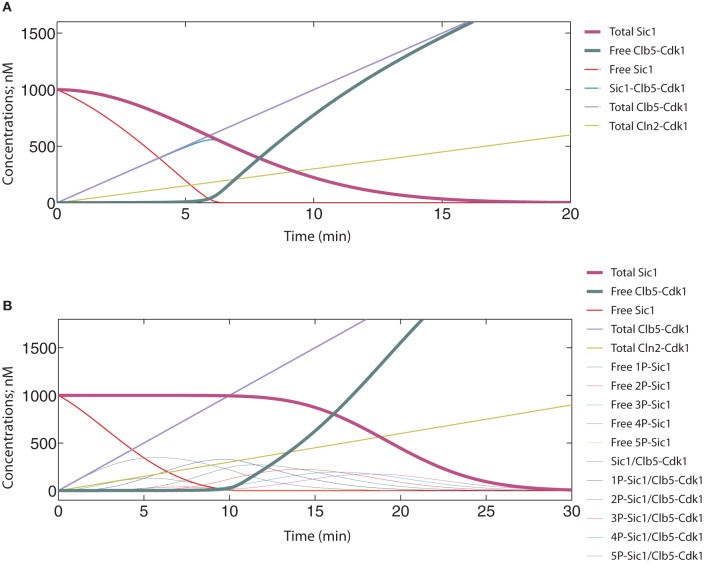
**Time course simulations for systems where only Cln2 is responsible for phosphorylation and degradation of Sic1. (A)** A model similar to that in Figures 8 and 9 was used, except that the rate constant value for Clb5-Cdk1 toward Sic1 was taken to be zero. The rate constant for Cln2-Cdk1 was 0.001 nM^−1^ × min^−1^, which is the same value used for Clb5-Cdk1 in Figure 8A. **(B)** A system that requires Cln2-dependent multisite phosphorylation for degradation of Sic1. The sequential phosphorylation of six sites in Sic1 was taken to be the output signal for degradation of Sic1.

We also tested the situation in which Cln2-Cdk1 alone was the kinase responsible for degradation of Sic1, and Clb5-dependent positive feedback was omitted (k_Clb5_ = 0). The abruptness of the switch was considerably reduced and the whole transition followed a path of a slow and gradual change (Figure 10A). This strongly suggests that the optimal mechanistic basis and the source of the ultrasensitive signal response of the switch would be the double-negative feedback effect of Clb5. If Cln1,2 were to act alone, the transition would not be sufficiently abrupt and there is no guarantee that it would not happen prematurely with insufficient Clb5 signal to promote S-phase. However, an earlier model of Sic1 phosphorylation and degradation based the responsibility of Sic1 phosphorylation entirely on Cln2 (Nash et al., 2001; Verma et al., 1997a). The ultrasensitive feature of the switch in this model was later built on a theoretical presumption of equal rates of distributive phosphorylation and the delayed accumulation of multiply phosphorylated forms of Sic1 (Deshaies and Ferrell, 2001). To test if this scheme would improve the abruptness of the switch, we introduced six equally Cln2-specific phosphorylation steps into the model, with phosphorylation at all six being required for degradation. Clb5-dependent positive feedback effect was excluded as in Figure 10A (for other details of this model see Table 3). The simulation of this system did indeed create a longer lag for the onset of the transition, but it did not improve the abruptness of the response (Figure 10B). In reality, the path of the transition would look even less switch-like because the N-terminus of Sic1 contains several crucial phosphorylation sites bearing suboptimal CDK consensus motifs and exhibits low individual site specificity with Cln2-Cdk1 (Koivomagi et al., 2011a). These simulations predict that although the delayed accumulation of multiply phosphorylated forms could create a threshold for Sic1 degradation, multisite phosphorylation would not add considerably to the abruptness of the switch. This conclusion is in agreement with the results of a theoretical analysis predicting that multisite phosphorylation may provide a threshold for a transition but is generally a poor switch (Gunawardena, 2005).

**Table 3:** **Equation system used for simulation of the time course for Cln2-dependent multisite phosphorylation of Sic1presented in Figure 10B**.

**Scheme**
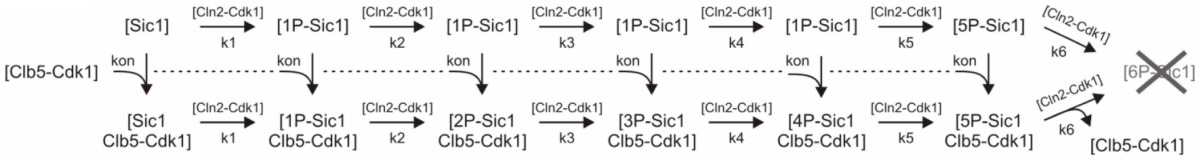
**The kinetic constants**
kon = 0.1;—the association rate of the inhibitory complex. To lower the complexity, the dissociation rate was considered very slow and not included in this version of the model.
*k*_1_ = 0.001; *k*_2_ = 0.001; *k*_3_ = 0.001; *k*_4_ = 0.001; and *k*_5_ = 0.001;—the rate constants for Cln2-dependent phosphorylation steps.
*k*_6_ = 0.001;—the rate constant for Cln2-dependent phosphorylation of 5P-Sic1 and 5P-Sic1/Clb5-Cdk1 including the net rate for the subsequent fast SCF-mediated degradation.
*kN*_1_ = 30; *kB*_1_ = 100; the synthesis rates of Cln2 and Clb5, respectively.
**The variables**
*y*_(1)_; *y*_(2)_; *y*_(3)_; *y*_(4)_; *y*_(5)_; *y*_(6)_;—Sic1 forms bearing 0, 1, 2, …, or 5 phosphates, respectively. The form with 6 phosphates was degraded by SCF and due to the fast rate of degradation this form was not expressed as a separate variable. To lower the complexity, only a sequential 6-step process was considered in the model.
*y*_(7)_; *y*_(8)_; *y*_(9)_; *y*_(10)_; *y*_(11)_; *y*_(12)_;—different Sic1/Clb5-Cdk1 inhibitory complexes involving Sic1 with 0, 1, 2, …, or 5 phosphates, respectively.
*y*_(13)_; *y*_(14)_; *y*_(15)_;—concentrations of free Clb5-Cdk1, Cln2-Cdk1, and total Clb5-Cdk1, respectively.
The initial value for 0P-Sic1 (*y*_(7)_) was taken to be 1, the initial values for the rest of the variables were taken to be zero.
**The ODE system**
*dy*_(1)_/*dt* = −*k*_1_ × *y*_(14)_ × *y*_(1)_ − kon × (*y*_(15)_ − *y*_(7)_ − *y*_(8)_ − *y*_(9)_ − *y*_(10)_ − *y*_(11)_ − *y*_(12)_) × y_(1)_
*dy*_(2)_/*dt* = *k*_1_ × *y*_(14)_ × *y*_(1)_ − *k*_2_ × *y*_(14)_ × *y*_(2)_ − kon × (*y*_(15)_ − *y*_(7)_ − *y*_(8)_ − *y*_(9)_ − *y*_(10)_ − *y*_(11)_ − *y*_(12)_) × *y*_(2)_
*dy*_(3)_/*dt* = *k*_2_ × *y*_(14)_ × *y*_(2)_ − *k*_3_ × *y*_(14)_ × *y*_(3)_ − kon × (*y*_(15)_ − *y*_(7)_ − *y*_(8)_ − *y*_(9)_ − *y*_(10)_ − *y*_(11)_ − *y*_(12)_) × *y*_(3)_
*dy*_(4)_/*dt* = *k*_3_ × *y*_(14)_ × *y*_(3)_ − *k*_4_ × *y*_(14)_ × *y*_(4)_ − kon × (*y*_(15)_ − *y*_(7)_ − *y*_(8)_ − *y*_(9)_ − *y*_(10)_ − *y*_(11)_ − *y*_(12)_) × *y*_(4)_
*dy*_(5)_/*dt* = *k*_4_ × *y*_(14)_ × *y*_(4)_ − *k*_5_ × *y*_(14)_ × *y*_(5)_ − kon × (*y*_(15)_ − *y*_(7)_ − *y*_(8)_ − *y*_(9)_ − *y*_(10)_ − *y*_(11)_ − *y*_(12)_) × *y*_(5)_
*dy*_(6)_/*dt* = *k*_5_ × *y*_(14)_ × *y*_(5)_ − *k*_6_ × *y*_(14)_ × *y*_(6)_ − kon × (*y*_(15)_ − *y*_(7)_ − *y*_(8)_ − *y*_(9)_ − *y*_(10)_ − *y*_(11)_ − *y*_(12)_) × *y*_(6)_
*dy*_(7)_/*dt* = kon × (*y*_(15)_ − *y*_(7)_ − *y*_(8)_ − *y*_(9)_ − *y*_(10)_ − *y*_(11)_ − *y*_(12)_) × *y*_(1)_ − *k*_1_ × *y*_(14)_ × (*y*_(15)_ − *y*_(13)_ − *y*_(8)_ − *y*_(9)_ − *y*_(10)_ − *y*_(11)_ − *y*_(12)_)
*dy*_(8)_/*dt* = kon × (*y*_(15)_ − *y*_(7)_ − *y*_(8)_ − *y*_(9)_ − *y*_(10)_ − *y*_(11)_ − *y*_(12)_) × *y*_(2)_ + (*k*_1_ × *y*_(14)_) × (*y*_(15)_ − *y*_(13)_ − *y*_(8)_ − *y*_(9)_ − *y*_(10)_ − *y*_(11)_ − *y*_(12)_) − (*k*_2_ × *y*_(14)_) × (*y*_(15)_ − *y*_(13)_ − *y*_(7)_ − *y*_(9)_ − *y*_(10)_ − *y*_(11)_ − *y*_(12)_)
*dy*_(9)_/*dt* = kon × (*y*_(15)_ − *y*_(7)_ − *y*_(8)_ − *y*_(9)_ − *y*_(10)_ − *y*_(11)_ − *y*_(12)_) × *y*_(3)_ + (*k*_2_ × *y*_(14)_) × (*y*_(15)_ − *y*_(13)_ − *y*_(7)_ − *y*_(9)_ − *y*_(10)_ − *y*_(11)_ − *y*_(12)_) − (*k*_3_ × *y*_(14)_) × (*y*_(15)_ − *y*_(13)_ − *y*_(8)_ − *y*_(7)_ − *y*_(10)_ − *y*_(11)_ − *y*_(12)_)
*dy*_(10)_/*dt* = kon × (*y*_(15)_ − *y*_(7)_ − *y*_(8)_ − *y*_(9)_ − *y*_(10)_ − *y*_(11)_ − *y*_(12)_) × *y*_(4)_ + (*k*_3_ × *y*_(14)_ × (*y*_(15)_ − *y*_(13)_ − *y*_(8)_ − *y*_(7)_ − *y*_(10)_ − *y*_(11)_ − *y*_(12)_) − (*k*_4_ × *y*_(14)_) × (*y*_(15)_ − *y*_(13)_ − *y*_(8)_ − *y*_(9)_ − *y*_(7)_ − *y*_(11)_ − *y*_(12)_)
*dy*_(11)_/*dt* = kon × (*y*_(15)_ − *y*_(7)_ − *y*_(8)_ − *y*_(9)_ − *y*_(10)_ − *y*_(11)_ − *y*_(12)_) × *y*_(5)_ + (*k*_4_ × *y*_(14)_) × (*y*_(15)_ − *y*_(13)_ − *y*_(8)_ − *y*_(9)_ − *y*_(7)_ − *y*_(11)_ − *y*_(12)_) − (*k*_5_ × *y*_(14)_) × (*y*_(15)_ − *y*_(13)_ − *y*_(8)_ − *y*_(9)_ − *y*_(10)_ − *y*_(7)_ − *y*_(12)_)
*dy*_(12)_/*dt* = kon × (*y*_(15)_ − *y*_(7)_ − *y*_(8)_ − *y*_(9)_ − *y*_(10)_ − *y*_(11)_ − *y*_(12)_) × *y*_(6)_ + (*k*_5_ × *y*_(14)_) × (*y*_(15)_ − *y*_(13)_ − *y*_(8)_ − *y*_(9)_ − *y*_(10)_ − *y*_(7)_ − *y*_(12)_) − (*k*_6_ × *y*_(14)_+) × (*y*_(15)_ − *y*_(13)_ − *y*_(8)_ − *y*_(9)_ − *y*_(10)_ − *y*_(11)_ − *y*_(7)_)
*dy*_(13)_/*dt* = *kB*_1_ − kon × (*y*_(15)_ − *y*_(7)_ − *y*_(8)_ − *y*_(9)_ − *y*_(10)_ − *y*_(11)_ − *y*_(12)_) × (*y*_(1)_ + *y*_(2)_ + *y*_(3)_ + *y*_(4)_ + *y*_(5)_ + *y*_(6)_) + *k*_6_ × *y*_(14)_ × *y*_(12)_
*dy*_(14)_/*dt* = *kN*_1_
*dy*_(15)_/*dt* = *kB*_1_

## Discussion

The double-negative feedback model of the G1/S switch in budding yeast involves two CDK complexes and a CDK inhibitor Sic1 that, at the same time, is the substrate of CDK. We aimed to explore which properties such a mutual antagonism would require to generate a useful switch. Based on mathematical modeling and phosphorylation assays we found that, for the transition to exhibit the best switch-like qualities, it is important to have tight inhibition of Clb5-Cdk1 by Sic1 and efficient phosphorylation of the Sic1-Clb5-Cdk1 inhibitory complex. The low K_*i*_ ensures that a tight and nearly stoichiometric barrier sets a threshold for the Clb5 signal. The efficient phosphorylation and degradation rates ensure that the double-negative feedback would gain the momentum required to create a switch-like increase in free Clb5-Cdk1 in response to total Clb5. We also studied the relative impact and different roles of Cln2 and Clb5 in the switch. In this respect we analyzed several possible scenarios: first, a scenario in which both Cln2 and Clb5 are capable of independently triggering the degradation of Sic1; second, a case in which Cln2 phosphorylates Sic1 to prime further Cks1-dependent phosphorylation by Clb5 but is not able to cause the degradation of Sic1 by itself; third, we modeled the situations in which either Cln2 or Clb5 alone was responsible for phosphorylation and degradation of Sic1; finally, we explored the impact of multisite phosphorylation on the switch. The general conclusion derived from these case studies was that the optimal role for Cln2-Cdk1 in the switch would not be to act as a kinase activity responsible for abrupt degradation of CKI, but rather to act as a priming signal that triggers the feedback loop for the emerging free Clb5-Cdk1.

Clb5-dependent positive feedback would be predicted to play a role in Sic1 degradation at the G1/S transition, since there is expected to be a point when the accumulating fraction of free Clb5-Cdk1 will overcome and phosphorylate Sic1 molecules that are bound to the inhibitory complex. This feedback is clearly expected to have some contribution to the abruptness of the switch. However, until recently, the extent of this effect had not been quantitatively estimated. In addition to our recent findings, Tang and colleagues used live cell fluorescent microscopy to show that Clb5,6-dependent positive feedback promotes the switch-like properties of the G1/S transition (Tang, pers. communication). Additionally, recent work by Barberis and colleagues has shown the involvement of Clb5 and also later B-type cyclins in degradation of Sic1 (Barberis et al., 2012). The authors of this work proposed a model where a surviving fraction of Sic1 could be involved even post-G1/S in the regulation of transcriptional waves of later cyclin genes. In general, these findings further argue against a solely Cln2-dependent mechanism of Sic1 degradation and emphasize the relatively major importance of B-type cyclins in this process.

The mutual control between Clb5 and Cln2 in tuning the threshold point and preventing a premature G1/S transition emphasizes the advantage of Cln2 as a primer rather than as a qualitatively similar kinase signal relative to Clb5. In addition, we have found that there could be other kinases besides Cln1,2 involved in priming of Sic1 for Clb5, since some phosphorylation of an N-terminal site is seen in G1-arrested cells (Koivomagi et al., 2011a). The inability of Cln1,2 to degrade Sic1 *in vivo* would likely also be partly caused by the much lower nuclear concentration of Cln2 compared to Clb5. Thus, it is possible that Cln1,2-Cdk1s play only a minor role in the switch.

The initial idea that Cln1,2-Cdk1 alone could be responsible for degradation of Sic1 was mainly based on an experiment in which about 2-fold overexpression of the non-degradable form of Sic1, relative to the endogenous levels of Sic1, did not inhibit endogenous Sic1 degradation but was able to block the start of replication (Verma et al., 1997a). However, our recent experiment showed that about 4-fold overexpression (our unpublished quantifications) of the non-degradable inhibitory domain of Sic1 fully stabilized endogenous Sic1 (Koivomagi et al., 2011a). Thus, it seems that at relatively low levels of the inhibitor the active Clb5-Cdk1 is able to accumulate to the levels that exceed the threshold capable of degrading Sic1, but not to the levels required to initiate S-phase. Another study reported that when non-degradable Sic1 accumulates to about the same levels as endogenous wild-type Sic1, cells are still capable of initiating DNA replication with only a minor delay (Cross et al., 2007). It seems that if the barrier set by the non-degradable inhibitor is at the same levels as wild-type Sic1, then Clb5 will accumulate and can still trigger S-phase. If the barrier is about 2-fold, it can degrade Sic1 but cannot start S-phase. This raises the interesting possibility that the Clb5-Cdk1 threshold level for initiation of Sic1 degradation is lower than the threshold for triggering S-phase by phosphorylation of Sld2 and Sld3. This would ensure that the transition always starts before the initiation of replication. Interestingly, a similar, highly resolved substrate phosphorylation order has been demonstrated for the accumulation of mitotic cyclin-CDK activity in both budding yeast (Oikonomou and Cross, 2011) and human cells (Deibler and Kirschner, 2010; Gavet and Pines, 2010).

The minimal model introduced in this study could serve as a core system that could help to understand G1/S control modules in other organisms. The crucial CKI at the G1/S transition in human cells, the protein p27Kip1 (Hengst et al., 1994; Polyak et al., 1994; Toyoshima and Hunter, 1994), must be phosphorylated by CDK to be recognized by the SCF ubiquitin ligase for subsequent degradation by the proteasome pathway (Pagano et al., 1995). It is believed that a positive feedback effect is created by CyclinE-Cdk2-dependent phosphorylation of the phosphodegron residue at Thr187 (Sheaff et al., 1997). Furthermore, the phosphorylation of Thr187 is facilitated by priming phosphorylation at Tyr88 by tyrosine kinases of the extracellular mitogen initiated signaling pathways (Chu et al., 2007; Grimmler et al., 2007; Jakel et al., 2012). The priming grants CDK the access to Thr187 and partly activates the inhibited CyclinE/Cdk2 by loosening the inhibitory interaction. In fact, human tyrosine kinases, by transmitting growth factor or cytokine signals, and Cln1,2-Cdk1 in yeast, by transmitting the signal of the cell-size control system, both play similar roles in the cell cycle by initiating S-phase. In both of these cases, the priming activity is not inhibited by CKI. Thus, it is possible that the variations of the proposed priming mechanism coupled to the mutual negative feedback of the CKI/CDK system could be functionally conserved across species. Therefore, the modeling approach presented here to estimate kinetic parameters that influence the abruptness and irreversibility of the G1/S switch may provide general insight into G1/S control in humans. This knowledge is especially important since perturbations in the regulatory module governing the G1/S is widely observed in cancer (Chu et al., 2008).

## Methods

### Protein purification

The TAP method was applied for purification of cyclin-Cdk1 complexes as described previously for Clb5-TAP-Cdk1 (Puig et al., 2001; Ubersax et al., 2003). 3HA-Cln2-Cdk1 was purified according to published protocols (McCusker et al., 2007) using rabbit polyclonal antibody against the haemagglutinin epitope (Labas). N-terminal 6His-tagged recombinant Sic1wt and Sic1ΔC proteins were purified by cobalt affinity chromatography. Cks1 was purified as described in Reynard et al. (2000). Protein concentrations were measured by colloidal coomassie G-250 using BSA as a standard.

### Kinase assays

The general composition of the kinase assay mixture was as follows: 50 mM HEPES pH 7.4, 5 mM MgCl_2_, 150 mM NaCl, 0.1% NP-40, 20 mM imidazole, 2% glycerol, 2 mM EGTA, 0.2 mg/ml^−1^ BSA, 500 nM Cks1, and 500 μM ATP [with added [γ−^32^P]ATP (Perkin Elmer)]. For the inhibition assay twelve reactions with Sic1wt concentrations ranging from 10 to 0.04 nM and two reactions containing no inhibitor were used. As a reference substrate, bovine histone H1 (Upstate) with final concentration 2.5 μM was used. The concentration of kinase complex in the assay was 1.5–2 nM. Before starting the reaction enzyme-inhibitor complex was formed (2 min). Reaction was started by adding H1. Two timepoints, 7 and 14 min, were collected and analyzed. For the phosphorylation assays presented in Figure 2, 15 nM Sic1wt or Sic1ΔC and 30 nM kinase complex were used. Four time-points were collected: 20s, 40s, 60s, and 80s. For demonstrating enzyme activity and Sic1 inhibition ratios 2.5 uM H1 was used as a substrate. For data analysis GraphPad Prism 5.0 software was used.

### Modeling

The models were constructed based on mass action kinetics using values of the kinetic constants derived from biochemical experiments. Because Sic1 concentrations in the model were several times below the K_M_ values for Cdk1-dependent phosphorylation of Sic1, velocities were expressed using k_cat_/K_M_ as a second order rate constant (v_0_ = k_cat_/K_M_[S][E]) instead of the Michaelis–Menten equation. The kinetic schemes of the models are presented in Figure 3 (for model simulations in Figures 4–7) and Table 3 (for model simulations in Figure 10B). The time course simulations in Figures 8, 9, and 10A were based on the scheme in Figure 3 with the exceptions that the synthesis and the basal degradation rates of Sic1 were omitted and linear synthesis rates for Cln2 and Clb5 were added. For the steady state diagrams we solved the equation system using MATLAB and the steady state values for plotting the diagrams were calculated with a step of 0.01 of the total Clb5 levels. The time course simulations for Figures 4–10 were performed using the MATLAB ode45 solver.

### Conflict of interest statement

The authors declare that the research was conducted in the absence of any commercial or financial relationships that could be construed as a potential conflict of interest.

## Research Topic:

From structural to molecular systems biology: experimental and computational approaches to unravel mechanisms of kinase activity regulation in cancer and neurodegeneration

## Topic Editor:

Matteo Barberis, Humboldt University Berlin, Germany; Max Planck Institute for Molecular Genetics, Berlin, Germany
